# Couple Relationship and Parent-Child Relationship Quality: Factors Relevant to Parent-Child Communication on Sexuality in Romania

**DOI:** 10.3390/jcm8030386

**Published:** 2019-03-19

**Authors:** Meda Veronica Pop, Alina Simona Rusu

**Affiliations:** School of Psychology and Education Sciences, Babes-Bolyai University, 400029 Cluj-Napoca, Romania

**Keywords:** sexual communication anxiety, sexual perfectionism, parent-child communication, risky sexual behavior

## Abstract

This study of parents in Romania explores how perceptions of their couple relationship quality and of factors associated with it (such as sexual communication anxiety and sexual perfectionism) were related to their perception of aspects describing parenting dimensions relevant to the sexual education and sexual health of their children. The hypotheses tested in this study were supported by the data collected from 106 participants (aged 25 to 51 years), parents of 1 to 3 children: (1) sexual communication anxiety with one’s partner (but not sexual perfectionism) is a significant predictor for parents’ self-efficacy, outcome expectancy and communication and parenting behavior related to sexuality education; (2) parents’ self-efficacy and outcome expectancy about parent-child communication on sexual topics (including involvement in risky sexual behaviors) predict the level of parenting behavior in this respect; (3) parents’ sexual communication anxiety (but not their sexual perfectionism) together with their self-efficacy and outcome expectancy regarding parent-child communication about sexuality predict the level of parental sexuality-communication-and-education behavior.

## 1. Introduction

Available data from most parts of world indicate that young people are often lacking competencies and are erroneously or partially informed about sexuality, sexual health and sexual risk behavior, and that they are the population that is at the highest risk of negative outcomes associated with sexual health, but the literature also indicates that many of these aspects could be overcome through effective sexuality education programs and interventions [1,2,3]. Thus, improving or optimizing sexual health in young people should and oftentimes does constitute a priority for families and care-givers, local communities, states and global society.

There is a considerable need expressed and identified for successful sexuality education programs and interventions, both formal and informal, for young people and for parents, given the costs and consequences of a lack of competencies and of risky sexual behavior in young people [4]. In line with this, identifying psychosocial factors relevant to the quality of the parent-child relationship and thus for the sexuality education and sexuality communication behavior between parents and their children is a promising line of research [5,6,7].

The quality of a couple’s relationship and their perception of it could influence a number of aspects of the parent-child relationship [8] and vice versa [9,10]. Kouros and colleagues [11] found a positive association between daily evaluations of the emotional quality of a parent’s intimate/couple relationship and that of the parent-child relationship after controlling for relationship satisfaction and conflict and for parenting levels [11]. This *spillover effect* [10,11,12], that is the transfer of a person’s (particularly negative) affect, mood and behavior from one context to another or from one interaction to another, could be bidirectional [10,11]. The *compensation hypothesis* proposes that a compensation of negative aspects of the couple relationship might translate into a person investing parenting resources (time, attention, knowledge) and positive affect into their parent-child relationship [13]. The two models should not necessarily be mutually exclusive [11]. Studies investigating the influence that the quality of parent-child relationship might have on the parents’ couple relationship or the bi-directionality of these influences have found support for both hypotheses [9,10,14].

Empirical evidence exists highlighting the (primary and secondary) effect that some parenting interventions might have on childrens’ behavior, on the parent-child relationship and also on the couple relationship [9]. Also, it appears that mothers might be less vulnerable than fathers to the spillover effect from the couple relationship into the parent-child relationship [14].

Parents’ concern over their communication with their children on sexuality topics is an aspect commonly addressed by parental programs and interventions (as a means or a goal) due to communication’s intrinsic role in parent-child relationships [15,16]. Studies investigating parental *connectedness* [17] with its component parent-child (sexual) communication, found communication (and connectedness) to be playing a protective role against certain sexual risk behavior in which young people might engage [18,19].

Communication on sexual topics between adolescents and parents predicted adolescents’ sexual communication with their partners on similar topics and for the sexually active ones it predicted the use of protection during sex (such as condoms) [20]. Although some parents express fear of the possibility that communication about sexuality might cause adolescents and young people to start their sex lives earlier or increase the chances of them engaging in particular sexual behavior, data generally does not support this association [15,19,21,22].

The majority of parents report they wish to communicate “openly” with their children on this subject [23], although data indicates that many of the adolescents perceive their communication on various sexuality issues with their parents to be less than satisfactory [22]. Generally, mothers tend to communicate more (frequently and diversely) than fathers about sexuality and more with their daughters than with their sons [24]. Also, there is a similar discrepancy with regard to parent-child sexuality communication related outcomes (e.g., sexually protective behavior) in favor of girls/daughters [15]. Widman and colleagues [15] suggest that besides other factors associated with the parent-child relationship, the quality of the parents’ couple relationship might interact with the parent-child communication and with its effects on children and young people’s sexual behavior [15].

The perceived self-efficacy and outcome expectancies (both in parents and in young people) about certain sexuality and sexuality education behaviors and outcomes were identified as good predictors for the level of sexually protective behavior in which young people engage and for their intentions in that sense [25,26,27]. Perceived self-efficacy is a person’s beliefs and expectations of their capacity to successfully follow a certain behavior while outcome expectancy is the person’s beliefs regarding the likelihood of a particular behavior to produce a certain outcome [28].

The sexuality (education) and sexual health of young people with intellectual or developmental disabilities has not been the subject of many research efforts thus far [29]. Significantly fewer aspects of the association between couple relationship factors and parent-child relationship factors in parents and their children with developmental problems or difficulties have been investigated. In comparison to others, these parents experience higher levels of stress and lower levels of couple relationship satisfaction [30,31].

Although the literature on the subject is not extensive, it is known that young LGBT people and their parents face various additional and specific challenges regarding sexuality education, sexual health and general well-being [32]. Research efforts in the health promotion and prevention of risk behavior in sexual and gender minorities revealed that positive parenting practices, acceptance and support from families, and communication between parents and LGBT youth were found to have protective roles for young people’s health and well-being [32].

There is very little research in the area of sexual risk behavior and sexuality education with participants, young or otherwise, from Romania. Romania does not have sexuality education in the national curriculum; currently, it lacks a national strategy and has had inconsistent or partially successful public policies regarding sexual and reproductive health. Data provided by reports from various international health promotion organizations have, in recent years, placed Romania in undesired leading positions among European countries with respect to various sexual and reproductive health outcomes [33].

This study aims to explore the ways in which, for parents in Romania, the perception of their couple-relationship quality and of several factors associated with it (such as sexual communication anxiety and sexual perfectionism) is related to the perception of factors describing parenting dimensions relevant for the sexuality education of children and young people. The perception of the quality of the couple relationship was previously, in studies [34] of adult participants from Romania, associated with their perception of the quality of their sexual relationship, with their anxiety to talk about sexual issues with their partners, and with aspects of their sexual perfectionism. Sexual communication anxiety is the anxiety or fear associated with a real or anticipated communication with one’s sexual partner about sexuality [35]. Perfectionism is defined as a person’s constant striving to avoid mistakes (*flawlessness*), their establishing extremely high standards of performance, accompanied by a tendency to make excessively critical self-evaluations and to be preoccupied with others’ negative evaluations of them [36]. Sexual perfectionism refers to the perfectionistic beliefs, standards and expectations people have for sexual performance and relationships, i.e., perfectionism related to the sexual aspects of a relationship [37,38].

Thus, the following hypotheses were tested: (1) Sexual communication anxiety and sexual perfectionism are significant predictors (individually and together) for parents’ self-efficacy, outcome expectancy and communication-and-parenting behavior regarding sexuality education; (2) Parents’ self-efficacy and outcome expectancy about parent-child communication on sexual topics are predictors (separately and together) of the level of parenting behavior in this respect; and (3) Parents’ sexual perfectionism and sexual communication anxiety together with their self-efficacy and outcome expectancy regarding parent-child communication about sexuality predict the level of parental sexuality-communication-and-education behavior.

## 2. Experimental Section

The research design was non-experimental, correlational and predictive (with an exploratory component), with five variables: (1) sexual communication anxiety (SCA), (2) multidimensional sexual perfectionism (MSP), (3) parental self-efficacy about communicating with children about sexuality (SESC), (4) parental sexuality-education-and-communication behavior (SECB) and (5) parental outcome expectancy about communicating with children about sexuality (OECS).

### 2.1. Participants and Procedure

Data were collected online from a convenience sample (“chain” selection, [39]) of N = 106 participants from various regions in Romania between April and June 2017. The participants were aged between 25 and 51 years (M = 37.83 years, SD = 5.99). A percentage of 92.5% of them were women; 76.4% of the participants were married, 16% divorced, 5.7% were unmarried but in a relationship and 1.9% were single at that time. For participants in a relationship at that time (98.1%), the mean duration of that relationship was M = 13.48 years (SD = 7.07). The mean duration of the participants’ longest relationship was 13.64 years (SD = 6.94). The mean number of participants’ sexual/romantic partners up to the study time was M = 4.86 (SD = 5.11). 96.4% of the participants had university degrees. 46 (43.4%) participants were raising 1 child, 56 (52.8%) were raising 2 children and 4 participants (3.8%) were parents to 3 children. The mean age of the 170 children raised by the study participants was M = 8.34 years (SD = 5.54).

The selection was based on a single criterion: participants had to be parents (legal guardians) of at least one child (younger than 18 years) at the moment of the study. The survey was completed anonymously online on the www.esurveycreator.com platform. General research ethics prescriptions were followed, as well as the regulations on Research Ethics of Babes-Bolyai University (informed consent, confidentiality and anonymity of the data).

### 2.2. Instruments

(1) Multidimensional Sexual Perfectionism Questionnaire (MSPQ) [37,38] for MSP, (2) Sexual Communication Apprehension Items (SCAI) [35] for SCA; (3) Parenting and Child Sexuality Questionnaire (PCSQ) [40] for SESC and SECB; (4) and Parenting Outcome Expectancy Scale (POES) [41] for OECS. All measures were previously indicated by the literature to have had good psychometric qualities. Socio-demographic items were created for the purpose of this study (e.g., gender, educational background, professional status, relationship status, relationship lengths, number of lifetime partners, number of children, self-rated religiosity level).

Data analyses were performed with the Statistical Package for the Social Sciences (SPSS 17.0) program. Normality of score-frequency-distribution tests, correlation analyses and simple and multiple (hierarchical) linear regression analyses were conducted.

## 3. Results

The results (Spearman rho coefficients) of the correlation analyses on subscale scores of study measures can be seen in Table 1.

Table 1 shows significant Spearman rho correlation coefficients (*p* < 0.01, 2-tailed) of adequate values, describing the relation between global scores on OECS and PCSQ (*r_est_* = 0.628, *p* < 0.01), on OECS and SCAI (*r_s_* = −0.564, *p* < 0.01) and on PCSQ and SCAI (*r_s_* = −0.516, *p* < 0.01). MSPQ global scores had no statistically significant relation with global scores on other measures in the study, although the Spearman rho correlation coefficient’s value for the MSPQ and SCAI global scores almost reached statistical significance (*p* = 0.06, 2 -tailed). Of particular interest are PCSQ subscales 1 and 2, which assess two different variables of the study: the OECS scores significantly positively correlate with the PCSQ1-SE scores (*r_est_* = 0.657, *p* < 0.01) and with the PCSQ2-B scores (*r_s_* = 0.478, *p* < 0.01); the SCAI global scores significantly negatively correlate with the PCSQ1-SE scores (*r_s_* = −0.526, *p* < 0.01) and with the PCSQ2-B scores (*r_s_* = −0.391, *p* < 0.01) (see Table 1).

Regarding sexual perfectionism and its dimensions’ correlations with other variables of the study, the only statistically significant ones were between scores on: MSPQ2-PS and PCSQ1-SE (*r_est_* = −0.330, *p* < 0.01); MSPQ3-DP and PCSQ1-SE (*r_s_* = −0.215, *p* < 0.05); MSPQ5-PSD and OECS (*r_s_* = −0.245, *p* < 0.05), MSPQ5-PSD and PCSQ1-SE (*r_s_* = −0.392, *p* < 0.01); MSPQ5-PSD and SCAI (*r_s_* = 0.301, *p* < 0.01); and MSPQ global scores and PCSQ1-SE (*r_s_* = −0.300, *p* < 0.01) (see Table 1).

Simple linear regression analyses were carried out to test the predictor quality of some study variables as posited by hypotheses 1 and 2. Simple linear regression equations (df = 1 and residual df = 104) indicated that the following significant predictors were found: (1) the MSPQ5-PSD scores predicted the PCSQ1-SE scores (F = 12.557, *p* < 0.01; *R*^2^ = 0.108) and SCAI global scores (F = 11.384, *p* < 0.01 *R*^2^ = 0.099); (2) the SCAI global scores predicted the PCSQ1-SE scores (F = 39.982, *p* < 0.01, *R*^2^ = 0.278), PCSQ2-B scores (F = 22.244, *p* < 0.01, *R*^2^ = 0.176) and POES scores (F = 47.265, *p* < 0.01, *R*^2^ = 0.312); (3) the POES scores predicted the PCSQ1-SE scores (F = 81.050, *p* < 0.01, *R*^2^ = 0.438) and PCSQ2-B scores (F = 32.401, *p* < 0.01, *R*^2^ = 0.238) and (4) the PCSQ1-SE scores predicted the PCSQ2-B scores (F = 74.308, *p* < 0.01, *R*^2^ = 0.417) and POES global scores (F = 81.050, *p* < 0.01, *R*^2^ = 0.438).

Simple linear regression analyses were followed (when the case) by a multiple linear regression. For all regression models proposed, the data satisfactorily verified all the assumptions of a multiple regression analysis [39,42,43].

The regression equation found for the “predictors SCA and MSP-PSD and criterion SESC” model was significant: F(2,103) = 22.821, *p* < 0.000, with *R*^2^ = 0.307. The SESC predicted level was 220.912–0.658 (SCA) −1.280 (MSP-PSD), where 220.912 was the constant’s regression coefficient’s value. Only SCA predicted SESC significantly at a *p* < 0.01 level, but at *p* < 0.05 both predictors were significant.

The regression equation found for the “predictors OECS and SESC and criterion SECB” model was significant: F (2,103) = 37.782, *p* < 0.000, with *R*^2^ = 0.423. The SECB predicted level was −0.500 + 0.210 (SESC) + 0.151 (OECS), where −0.500 was the constant’s regression coefficient’s value. Only SESC was a significant predictor for SECB. The regression equation found for the “predictors OECS and SCA and criterion SESC” model was significant: F (2,103) = 46.381, *p* < 0.000, with *R*^2^ = 0.474. The SESC predicted level was 1.247–0.320 (SCA) + 2.062 (OECS), where 1.247 was the constant’s regression coefficient’s value. Both SCA and OECS were significant predictors for SESC.

The regression equation found for the “predictors SCA and SESC and criterion SECB” model was significant: F (2,103) = 38.144, *p* < 0.000, cu *R*^2^ = 0.426. The SECB predicted level was 15.114–0.056 (SCA) + 0.215 (SESC), where 15.114 was the constant’s regression coefficient’s value. Only SESC was a significant predictor for SECB.

**Figure 1:**
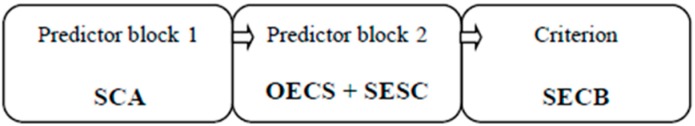
Hierarchical multiple regression model (Hypothesis 3 of study).

A two-step hierarchical regression analysis was carried out to test the third hypothesis of this study. One of the distal predictors (i.e., MSP) for the SECB criterion was excluded from the analysis due to the fact that previous analyses revealed that it was not a good predictor for the dependent variable of the model. As such, the first predictor block included only SCA as an independent variable while the second regression predictor block contained SESC and OECS (see Figure 1). Tests of the model data revealed that it met the assumptions of a multiple regression analysis.

The linear hierarchical (2-step) regression analysis returned significant (*p* < 0.001) regression equations for both models (steps): *model 1* (only predictor block 1) and *model 2* (predictor blocks 1 and 2) (see Table 2).

For *model 1*, the regression equation was F (1,104) = 22.244, *p* < 0.000, with *R*^2^ = 0.176. The level of the predicted SECB was 59.470–0.215 (SCA), where 59.470 was the constant’s regression coefficient value. For *model 2*, the regression equation was F (2,102) = 25.465, *p* < 0.000, with *R*^2^ = 0.428. The level of the predicted SECB was 7.573 - 0.045 (SCA) + 0.104 (OECS) + 0.201 (SESC), where 7.573 was the constant’s regression coefficient value (see Table 3). Both models contributed significantly (F value is significant, *p* < 0.000) to the capacity of predicting the criterion in comparison to models with estimated population parameters [44].

Both models explained a significant variance at the criterion level (see Table 2)*. Model 1* indicated that SCA significantly (*p* < 0.000) predicted the criterion SECB, i.e., 17.6% of its variance. *Model 2* indicated that together the three predictors (SCA, OECS and SESC) significantly (*p* < 0.000) predicted the criterion SECB, i.e., 42.8% of its variance. Thus, adding the two predictors (in block 2) to the hierarchical regression brought a significant (*p* < 0.000) improvement to the prediction model (R^2^_change_ = 0.252) of SECB. Adding OECS and SESC as predictors increased the percentage of criterion-variance prediction by 25.2% [44].

The values of the adjusted coefficient of determination (*R*^2^ *adjust.*) for both models of the hierarchical regression analysis were very similar to those of the coefficient of determination *R*^2^ (see Table 2), which indicates that if they were to be derived from the population and not from the study sample the two models of the hierarchical regression would explain approximately similar levels of the criterion variance. It could be thus said that the two models have a high generalizability level (Field, 2013).

Table 3 indicated that when SCA was the only independent variable in the model, it was a significant predictor for SECB (*t* = −4.716, *p* < 0.000), but once the other two predictors (OECS and SESC) were introduced in the regression analysis, SCA did not remain significant as a predictor of SECB (*t* = −0.950, *p* = 0.344). Also, OECS proved not to be a significant predictor for SECB when considered together with the other two predictors (*t* = 0.698, *p* = 0.487). In this model (i.e., 2) the only predictor that remained significant for the criterion variance was SESC (*t* = 5.328, *p* < 0.000). Thus, although the three predictors had, separately, a significant direct influence on the criterion (as shown by the results of simple regression analyses), when their interaction was taken into consideration (controlling for levels of any two of them), the only one retaining a significant direct influence on SECB in this model was SESC. SCA and OECS lost their influence in this model as direct predictors of SECB and only showed an indirect influence [44].

**Figure 2:**
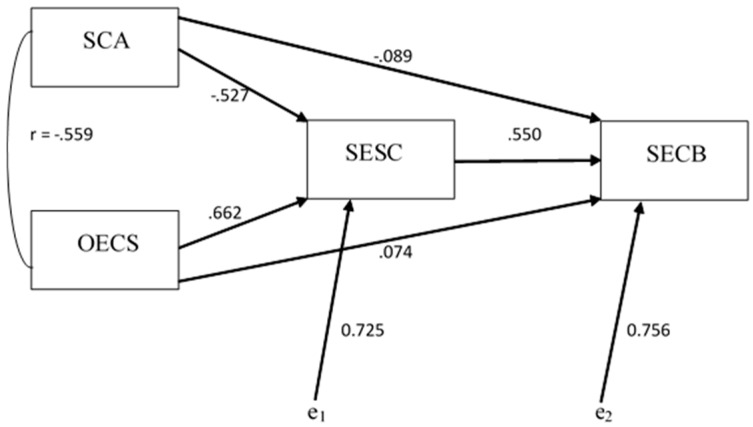
Mediation model of the relation between the predictors SCA, OECS and the criterion SECB by the predictor SESC.

Based on these results a mediation model was proposed with SESC mediating the relation/path between the predictors SCA and OECS with SECB. Figure 2 describes this model. The validity of this model needs further testing in future studies.

The information offered by the parents in Romania participating in the study based on their answers to the socio-demographic data questionnaire revealed that a percentage of 94.3% (N = 100) did not consider their children to have ever been in a sexual risk situation. Only 31.13% (N = 33) of them were able to describe what in their opinion could constitute such a situation (e.g., exposure to online pornography, unprotected sex or being approached for sex by strangers, adults or older children/young people). 23.6% (n = 25) of the 106 participants reported that they had never used any type of resources to help them with the communication about sexuality and sexuality education they provided to their children; the majority of the participants, 64.2% (n = 68), mentioned books as a source of information, while 56.6% (n = 60) of them mentioned online resources and 40.6% (n = 43) of them mentioned talking to friends. Only 17.9% (n = 19) had talked to professionals while 11.3% (n = 12) had attended a specific course/training. During the 6 months prior to the study, 35 (33%) participants did not use any type of resource, 27 (25.5%) participants used them rarely, another 27 participants used them moderately frequently, while only 14 (13.2%) used them quite frequently and 3 (2.8%) used them very frequently. 17.9% (n = 19) of participants had not communicated to their children about sexuality in the 6 months prior to the study, 27.4% (n = 29) had only communicated rarely, 32.1% (n = 34) had communicated moderately frequently, 21.7% (n = 23) quite often, and 0.9% (n = 1) reported communicating very often.

Participants’ self-rated level of religiosity was not a good predictor for any of the variables of the study. The number of sexual partners that participants estimated they had had by that time (*M* = 4.86, *SD* = 5.109) proved to be a moderate predictor for their level of self-efficacy regarding communication with children on sexuality topics and for their level of sexuality education parenting behavior. The majority of participants responded to the three optional open-ended questions in PCSQ [40] regarding communication about sexuality and sexuality education programs, i.e., all of the participants responded to the question “Please describe how you communicate with your child about sexuality?”, more than 99% of them responded to “What would make it easier for you to talk to your child about sexuality?” and 95% responded to “What additional information or topics would you like to see included in a parenting program to help parents develop skills to support children’s developing sexuality?”.

The responses to the first of these questions were very diverse, although participants predominantly answered that they communicated “openly” and in a “relaxed” way, and that they had “positive” and “natural” conversations on the topic with their children, many using, as expected, the verbal approach to communication. Only seven participants stated they didn’t communicate with their children about sexuality, citing mainly the child’s “inappropriate” age as a reason for it. Some parents (around 25%) used the only-answer-when-asked approach, while others (also approximately 25%) mentioned they initiated conversations. Many of them stressed the anatomic and physiological aspects of development in their conversations with their children and expressed beliefs about the “age-appropriateness” of the conversations’ content. One parent said “With honesty, trust and responsibility and without thinking my children are too young to know the truth. I tell them what they need to know at their age based on their cognitive development phase. I answer their questions about sexuality. We do not hide our bodies, we use the appropriate names for genitalia” while another said, “I haven’t talked to my children about this subject. I don’t feel prepared for such conversations. I don’t have the necessary courage to talk to them.”

To the second question, 13 participants responded with “I don’t know”, while 18 parents (17%) said schools (and also sometimes pre-schools) should provide sexuality education classes for children. Participants mostly mentioned the following means of facilitating communication with children about sexuality: being properly informed and trained; having access to various types of resources and support; changed societal and individual (their own) attitudes regarding sexuality; their children’s age and perceived interest for the topic. Almost a fifth of the participants considered that the communication with their children about sexuality was good and it couldn’t be improved. One parent said that “Sexuality education in schools using accurate scientific resources and leaving aside any unnecessary self-consciousness would help”, while another participant responded that ”It would be helpful to involve parents in having all the necessary knowledge to approach all the aspects of sexuality in a competent and relaxed way”.

To the third open-ended question, approximately one third of the participants responded by saying they wouldn’t add anything to a sexuality education parenting program besides the topics already mentioned in the previous item of PCSQ. Almost a quarter of the respondents considered that the parent-child relationship and communication about sexuality should be part of a parental sexuality education program, and a similar number of parents thought that such a program should include information on how to access accurate information sources for both parents and children and also information about children’s and adolescents’ development and about age-appropriate communication. Approximately 10% of the parents considered that information about negative consequences of sexual activity, sexually transmitted infections, protection, pregnancy and contraception, sexual orientation and gender identity, morality, religion and their relation to sexuality, should be added. A few parents mentioned that a sexuality education parenting program should also be about romantic relationships, consent, abuse, media influence and pornography. One participant said that “If we want a healthier generation, adopting older generations’ models will only bring negative consequences; as such, sexuality education should be provided by professionals and with minimum involvement from dilettantes in the subject, be they well-intended parents” and another noted, “How to communicate so that we don’t push them away from us and that they come and ask for advice when they need it, even in this sensitive domain. Children rarely talk to their parents about this subject”.

## 4. Discussion

The results of the investigation on parents from Romania support the fact that participants’ level of sexual communication (with their partner) anxiety predicted their level of parental outcome expectancy and self-efficacy regarding communication with children about sexuality, as well as the level of communication-with-children-about-sexuality behavior they engaged in.

With regard to the sexual perfectionism dimensions, partners’ self-directed (towards respondent) sexual perfectionism was found to be a significant predictor for respondents’ level of sexual communication anxiety and for their level of parental self-efficacy about discussing sexuality. Moreover, this dimension of sexual perfectionism proved to be significantly correlated with the majority of the study’s variables and their dimensions, with the exception of parental communication-about-sexuality-and-sexuality-education behavior. As a result of that, sexual perfectionism was replaced by this dimension (partners’ self-directed sexual perfectionism) throughout the following analyses of the study. Sexual communication anxiety and partners’ self-directed sexual perfectionism together significantly predicted the level of parental self-efficacy of communication with children about sexuality, sexual communication anxiety being a mediator in their relation. Other multiple prediction models were not tested due to the fact that partners’ self-directed sexual perfectionism was not a significant predictor for the other variables. Since no prior results on this subject (hypothesis 1) were found in literature, a comparison could not be made, but theoretical models and other connected results encouraged such a hypothesis being formulated and the attempt made in this direction by this study indicated promising results.

Regarding the second hypothesis of the study, the data analysis revealed that parental self-efficacy and outcome expectancy about communicating with children on topics of sexuality were significant predictors (both separately and together) for the parental level of communication about sexuality and sexual education with the children. Parents’ communication self-efficacy appeared to mediate the relation of the other two variables.

Both self-efficacy and outcome expectancy were good predictors for each other. When taking into account their interaction, only self-efficacy about communicating with children on sexuality topics remained significant in predicting the level of communication behavior between parents and children about sexuality. These results confirmed, on the one hand, the predictions of Bandura’s theory of self-efficacy regarding the role that self-efficacy and outcome expectancy played in predicting the performance and intention to perform certain behaviors. On the other hand, they partially contradicted Bandura’s view [28] of these processes, offering alongside other results [45] valuable insights about the possibility of a bi-causal relation existing between parental self-efficacy and outcome expectancy with regard to their communication with children on sexuality topics.

The third hypothesis of the study tested a two-step multiple prediction model for the level of parental communication-with-children-about-sexuality behavior. Sexual communication anxiety was a predictor in the first block of predictors and parental outcome expectancy and self-efficacy regarding communication with children about sexuality were in the second prediction block. The results of the model testing pointed out that only parental self-efficacy about communication with children on sexuality topics remained a significant predictor for their levels of parenting behavior in that respect. The other two predictors had only an indirect effect over the parental communication with children about sexuality. A path model describing these relations was built. These findings are among the very few results proposing a model that describes the relations between these variables (i.e., characterizing parents’ perceptions of their couple relationship and of their parental relationship and parenting aspects) with an explanatory value for the variance in the levels of parents’ communication-with-children-about-sexuality behavior and with implications both at a theoretical and a practical level.

From a practical point of view, these results have a potential applicability in the configuration of new or in the adjustment of already-existing family counselling interventions, as well as in educational approaches such as sexuality education programs addressed to young people and/or to their parents. Based on the explored model of prediction and mediation from this study, these interventions could target the perception of a couple’s sexual relationship or of the parent-child relationship with the projected outcome of changing the sexuality-communication behavior between parents and children while also bringing other secondary benefits in the parent-child relationship and also in the couple relationship. Specifically, these benefits refer to lowering the levels of anxiety about communication on sexual topics with one’s partner or the levels of one’s sexual perfectionism, which in turn could contribute to the quality of one’s intimate relationships. Understandably, these results, especially the ones obtained by testing exploratory hypotheses, need further investigation in future studies with the purpose of better comprehending this research area and the associations between individual characteristics, family dynamics and processes which influence the sexual health outcomes in young people.

There are some possible limitations to the conclusions drawn from the results of this study. Among them might be the characteristics of the study sample (e.g., mostly women, mostly married or in a long-term relationship, mostly holding a university degree), while others relate to the study procedure and the assessment instruments (e.g., access restricted to online participation, some of instruments translated but not validated), and others relate to the data sample. Our opinion is that these possible limitations affecting the generalizability of our conclusions could be seen as an opportunity and a basis for future studies, where their influence on the results could be additionally investigated and understood.

In conclusion, the study successfully explored and investigated how factors characterizing parents-from-Romania’s perceptions of their (sexual) couple relationships and of their parent-child relationships were both relevant for their communication with their children on sexual topics. The more anxious participating parents were about communicating about sexual issues with their partner and the less confident they were about their capacity to communicate with their children about sexuality or about the effects of such a communication, the less likely they were to talk with their children about sexuality.

## Figures and Tables

**Table 1:** Spearman bivariate correlation coefficients for study variables (and dimensions) and significance levels.

	1	2	3	4	5	6	7	8	9	10	11	12	13	14	15
1 POES total															
														
2 PCSQ1-SE	0.657 **														
	0.000														
3 PCSQ2-B	0.478 **	0.654 **													
	0.000	0.000													
4 PCSQ3-E	0.273 **	0.289 **	0.389 **												
	0.005	0.003	0.000												
5 PCSQ total	0.628 **	0.925 **	0.848 **	0.498 **											
	0.000	0.000	0.000	0.000											
6 MSPQ1-SO	0.072	−0.080	0.059	0.154	−0.019										
	0.462	0.414	0.545	0.115	0.849										
7 MSPQ2-SP	−0.149	−0.330 **	−0.068	0.060	−0.217 *	0.517 **									
	0.128	0.001	0.489	0.540	0.025	0.000									
8 MSPQ3-PD	−0.106	−0.215 *	−0.005	0.138	−0.117	0.713 **	0.468 **								
	0.280	0.027	0.958	0.159	0.231	0.000	0.000								
9 MSPQ4-PSO	−0.013	−0.234 *	−0.039	0.156	−0.120	0.556 **	0.443 **	0.568 **							
	0.894	0.016	0.690	0.109	0.219	0.000	0.000	0.000							
10 MSPQ5-PSD	−0.245 *	−0.392 **	−0.119	0.062	−0.279 **	0.510 **	0.596 **	0.704 **	0.627 **						
	0.011	0.000	0.225	0.528	0.004	0.000	0.000	0.000	0.000						
11 MSPQ total	−0.103	−0.300 **	−0.013	0.162	−0.170	0.793 **	0.716 **	0.852 **	0.775 **	0.854 **					
	0.291	0.002	0.896	0.098	0.081	0.000	0.000	0.000	0.000	0.000					
12 SCAI1-G	−0.547 **	−0.509 **	−0.402 **	−0.277 **	−0.507 **	0.029	0.180	0.146	0.059	0.283 **	0.174				
	0.000	0.000	0.000	0.004	0.000	0.769	0.065	0.135	0.550	0.003	0.074				
13 SCAI2-SS	−0.437 **	−0.475 **	−0.287 **	−0.219 *	−0.441 **	0.091	0.187	0.196 *	0.141	0.341 **	0.230 *	0.709 **			
	0.000	0.000	0.003	0.024	0.000	0.352	0.055	0.045	0.150	0.000	0.017	0.000			
14 SCAI3-ND	−0.536 **	−0.495 **	−0.359 **	−0.231 *	−0.483 **	−0.058	0.173	0.089	0.001	0.249 *	0.112	0.829 **	0.691 **		
	0.000	0.000	0.000	0.017	0.000	0.553	0.077	0.367	0.996	0.010	0.252	0.000	0.000		
15 SCAI total	−0.564 **	−0.526 **	−0.391 **	−0.283 **	−0.516 **	0.024	0.182	0.154	0.065	0.301 **	0.183	0.983 **	0.797 **	0.869 **	
	0.000	0.000	0.000	0.003	0.000	0.810	0.062	0.116	0.507	0.002	0.060	0.000	0.000	0.000	

** = level of significance *p* < 0.01 (2-tailed); * = level of significance *p* < 0.05 (2-tailed). Note: PCSQ subscales: PCSQ1-SE = Confidence and Comfort; PCSQ2-B = Parenting Behavior; PCSQ3-E = Sexuality Education; MSPQ subscales: MSPQ1-SO = Self-oriented sexual perfectionism; MSPQ2-SP = Socially prescribed sexual perfectionism; MSPQ3-PD = Partner-directed sexual perfectionism; MSPQ4-PSO = Partner’s self-oriented sexual perfectionism; MSPQ5-PSD = Partner’s self(respondent)-directed sexual perfectionism; SCAI subscales: SCAI1-G = General sexual communication anxiety; SCAI2-SS = Safer sex communication anxiety SCAI3-ND = Negative disclosure anxiety. Bolded characters indicate the category of statistical significant results.

**Table 2:** Parameters of the hierarchical regression models (model 1 and model 2).

Regression Model	Model Parameters					Change Parameters		
	*R*	*R* ^2^	*R*^2^ Adjust.	F	*p*	*R*^2^ Change	F Change	p_Fch_
1	0.420 ^a^	0.176	0.168	22.244	0.000 ^a^	0.176	22.244	0.000
2	0.654 ^b^	0.428	0.411	25.465	0.000 ^b^	0.252	22.481	0.000

*R* = correlation coefficient; *R*^2^ = determination coefficient; *R*^2^ adjust. = adjusted determination coefficient; F = global significance of predictor; *p* = level of significance; ^a^ Predictors: (Constant), SCA; ^b^ Predictors: (Constant), SCA, OECS, SESC; Criterion: SECB.

**Table 3:** Hierarchical regression coefficients (hypothesis 3 of the study).

Regression Model		Unstandard. Coeff.		Standard. Coeff.	*t*	*p*	95% Confidence Interval for B		Correlations		
		B	SE	β			Lower Limit	Upper Limit	Zero-Order	Partial	Semi-Partial
1	(Constant)	59.470	2.655		22.399	0.000	54.205	64.735			
	SCAI total	−0.215	0.046	−0.420	−4.716	0.000	−0.305	−0.124	−0.420	−0.420	−0.420
2	(Constant)	7.573	12.881		0.588	0.558	−17.976	33.123			
	SCAI total	−0.045	0.048	−0.089	−0.950	0.344	−0.140	0.049	−0.420	−0.094	−0.071
	POES total	0.104	0.149	0.074	0.698	0.487	−0.192	0.400	0.487	0.069	0.052
	PCSQ1-SE	0.201	0.038	0.550	5.328	0.000	0.126	0.276	0.646	0.467	0.399

B = regression coefficient/slope value; SE = coefficient standard error; β = standardized coefficient value; *t* = significance of coefficient test statistic; *p* = probability significance level.

## References

[1] Hirst J. (2008). Developing sexual competence? Exploring strategies for the provision of effective sexualities and relationships education. Sex Educ..

[2] United Nations Educational, Scientific and Cultural Organization (UNESCO) (2009). The Rationale for Sexuality Education. International Technical Guidance on Sexuality Education: An Evidence-Informed Approach for Schools, Teachers and Health Educators.

[3] Bourke A., Boduszek D., Kelleher C., McBride O., Morgan K. (2014). Sex education, first sex and sexual health outcomes in adulthood: Findings from a nationally representative sexual health survey. Sex Educ..

[4] Kirby D. (2011). Sex Education: Access and Impact on Sexual Behaviour of Young People.

[5] de Graaf H., Vanwesenbeeck I., Woertman L., Meeus W. (2011). Parenting and adolescents’ sexual development in western societies: A literature review. Eur. Psychol..

[6] Kelleher C., Boduszek D., Bourke A., McBride O., Morgan K. (2013). Parental involvement in sexuality education: Advancing understanding through an analysis of findings from the 2010 Irish Contraception and Crisis Pregnancy Study. Sex Educ..

[7] Stone N., Ingham R., Gibbins K. (2013). ‘Where do babies come from?’ Barriers to early sexuality communication between parents and young children. Sex Educ..

[8] Morrill M.I., Hawrilenko M., Córdova J.V. (2016). A longitudinal examination of positive parenting following an acceptance-based couple intervention. J. Fam. Psychol..

[9] Zemp M., Milek A., Davies P.T., Bodenmann G. (2016). Improved child problem behavior enhances the parents’ relationship quality: A randomized trial. J. Fam. Psychol..

[10] Sears M.S., Repetti R.L., Reynolds B.M., Robles T.F., Krull J.L. (2016). Spillover in the home: The effects of family conflict on parents’ behavior. J. Marriage Fam..

[11] Kouros C.D., Papp L.M., Goeke-Morey M.C., Cummings E.M. (2014). Spillover between marital quality and parent–child relationship quality: Parental depressive symptoms as moderators. J. Fam. Psychol..

[12] Stroud C.B., Meyers K.M., Wilson S., Durbin C.E. (2015). Marital quality spillover and young children’s adjustment: Evidence for dyadic and triadic parenting as mechanisms. J. Clin. Child Adolesc. Psychol..

[13] Nelson J.A., O’Brien M., Blankson A.N., Calkins S.D., Keane S.P. (2009). Family stress and parental responses to children’s negative emotions: Tests of the spillover, crossover, and compensatory hypotheses. J. Fam. Psychol..

[14] Khajehei M. (2015). Parenting challenges and parents’ intimate relationships. J. Hum. Behav. Soc. Environ..

[15] Widman L., Choukas-Bradley S., Noar S.M., Nesi J., Garrett K. (2016). Parent-adolescent sexual communication and adolescent safer sex behavior: A meta-analysis. JAMA Pediatr..

[16] Wight D., Fullerton D. (2013). A review of interventions with parents to promote the sexual health of their children. J. Adolesc. Health.

[17] Vidourek R.A., Bernard A.L., King K.A. (2009). Effective parent connectedness components in sexuality education interventions for African American youth: A review of the literature. Am. J. Sex. Educ..

[18] Markham C.M., Lormand D., Gloppen K.M., Peskin M.F., Flores B., Low B., House L.D. (2010). Connectedness as a predictor of sexual and reproductive health outcomes for youth. J. Adolesc. Health.

[19] De Looze M., Constantine N.A., Jerman P., Vermeulen-Smit E., ter Bogt T. (2015). Parent–adolescent sexual communication and its association with adolescent sexual behaviors: A nationally representative analysis in the Netherlands. J. Sex Res..

[20] Widman L., Choukas-Bradley S., Helms S.W., Golin C.E., Prinstein M.J. (2014). Sexual communication between early adolescents and their dating partners, parents, and best friends. J. Sex Res..

[21] Zamboni B.D., Silver R. (2009). Family sex communication and the sexual desire, attitudes, and behavior of late adolescents. Am. J. Sex. Educ..

[22] Angera J.J., Brookins-Fisher J., Inungu J.N. (2008). An investigation of parent/child communication about sexuality. Am. J. Sex. Educ..

[23] Kirkman M., Rosenthal D.A., Shirley Feldman S. (2005). Being open with your mouth shut: The meaning of ‘openness’ in family communication about sexuality. Sex Educ..

[24] Sneed C.D., Somoza C.G., Jones T., Alfaro S. (2013). Topics discussed with mothers and fathers for parent–child sex communication among African-American adolescents. Sex Educ..

[25] DiIorio C., Dudley W.N., Kelly M., Soet J.E., Mbwara J., Potter J.S. (2001). Social cognitive correlates of sexual experience and condom use among 13-through 15-year-old adolescents. J. Adolesc. Health.

[26] DiIorio C., McCarty F., Denzmore P. (2006). An exploration of social cognitive theory mediators of father–son communication about sex. J. Pediatric Psychol..

[27] Lehr S.T., Demi A.S., DiIorio C., Facteau J. (2005). Predictors of father-son communication about sexuality. J. Sex Res..

[28] Bandura A. (1977). Self-efficacy: Toward a unifying theory of behavioral change. Psychol. Rev..

[29] Sinclair J., Unruh D., Lindstrom L., Scanlon D. (2015). Barriers to sexuality for individuals with intellectual and developmental disabilities: A literature review. Educ. Train. Autism Dev. Disabil..

[30] Kersh J., Hedvat T.T., Hauser-Cram P., Warfield M.E. (2006). The contribution of marital quality to the well-being of parents of children with developmental disabilities. J. Intellect. Disabil. Res..

[31] Miodrag N., Hodapp R.M. (2010). Chronic stress and health among parents of children with intellectual and developmental disabilities. Curr. Opin. Psychiatry.

[32] Bouris A., Guilamo-Ramos V., Pickard A., Shiu C., Loosier P.S., Dittus P., Gloppen K., Waldmiller J.M. (2010). A systematic review of parental influences on the health and well-being of lesbian, gay, and bisexual youth: Time for a new public health research and practice agenda. J. Prim. Prev..

[33] Pop M.V., Rusu A.S. (2017). Developing a sexuality education program for parents in Romania–preliminary analysis. J. Psychol. Educ. Res..

[34] Pop M.V., Rusu A.S. (2015). Satisfaction and communication in couples of parents and potential parents—psychological predictors and implications for sexuality education of children. Procedia-Soc. Behav. Sci..

[35] Babin E.A. (2012). An examination of predictors of nonverbal and verbal communication of pleasure during sex and sexual satisfaction. J. Soc. Pers. Relationsh..

[36] Hewitt P.L., Flett G.L. (1991). Perfectionism in the self and social contexts: Conceptualization, assessment, and association with psychopathology. J. Personal. Soc. Psychol..

[37] Snell W.E. (2001). Chapter 16: Sexual perfectionism among single sexually experienced females. New Directions in the Psychology of Human Sexuality.

[38] Snell W.E., Rigdon K.L., Snell W.E. (2001). Chapter 15: The Multidimensional Sexual Perfectionism Questionnaire: Preliminary evidence for reliability and validity. New Directions in the Psychology of Human Sexuality.

[39] Clark-Carter D. (2010). Quantitative Psychological Research: The Complete Student’s Companion.

[40] Morawska A., Walsh A., Grabski M., Fletcher R. (2015). Parental confidence and preferences for communicating with their child about sexuality. Sex Educ..

[41] DiIorio C., Dudley W.N., Wang D.T., Wasserman J., Eichler M., Belcher L., West-Edwards C. (2001). Measurement of parenting self-efficacy and outcome expectancy related to discussions about sex. J. Nurs. Meas..

[42] Howitt D., Cramer D. (2017). Understanding Statistics in Psychology with SPSS.

[43] Tabachnick B.G., Fidell L.S. (2013). Using Multivariate Statistics.

[44] Field A. (2013). Discovering Statistics Using IBM SPSS Statistics.

[45] Williams D.M. (2010). Outcome expectancy and self-efficacy: Theoretical implications of an unresolved contradiction. Personal. Soc. Psychol. Rev..

